# High-Cycle Fatigue Life Prediction of Additive Manufacturing Inconel 718 Alloy via Machine Learning

**DOI:** 10.3390/ma18112604

**Published:** 2025-06-03

**Authors:** Zongxian Song, Jinling Peng, Lina Zhu, Caiyan Deng, Yangyang Zhao, Qingya Guo, Angran Zhu

**Affiliations:** 1 School of Aeronautics and Astronautics, Tianjin Sino-German University of Applied Sciences, Tianjin 300350, China; songzongxian@tsguas.edu.cn (Z.S.); a1728714534@163.com (J.P.); 17746189022@163.com (Q.G.); 18625570183@163.com (A.Z.); 2 Wheel Rail Center, Tianjin Research Institute for Advanced Equipment, Tsinghua University, Tianjin 300300, China; zhuln@tsinghua-tj.org; 3 School of Materials Science and Engineering, Tianjin University, Tianjin 300350, China; dengcy@tju.edu.cn

**Keywords:** fatigue life prediction, additive manufacturing, Inconel 718 alloy, machine learning

## Abstract

This study established a machine learning framework to enhance the accuracy of very-high-cycle fatigue (VHCF) life prediction in selective laser melted Inconel 718 alloy by systematically comparing the use of generative adversarial networks (GANs) and variational auto-encoders (VAEs) for data augmentation. We quantified the influence of critical defect parameters (dimensions and stress amplitudes) extracted from fracture analyses on fatigue life and compared the performance of GANs versus VAEs in generating synthetic training data for three regression models (ANN, Random Forest, and SVR). The experimental fatigue data were augmented using both generative models, followed by hyperparameter optimization and rigorous validation against independent test sets. The results demonstrated that the GAN-generated data significantly improved the prediction metrics, with GAN-enhanced models achieving superior R^2^ scores (0.91–0.97 vs. 0.86 ± 0.87) and lower MAEs (1.13–1.62% vs. 2.00–2.64%) compared to the VAE-based approaches. This work not only establishes GANs as a breakthrough tool for AM fatigue prediction but also provides a transferable methodology for data-driven modeling of defect-dominated failure mechanisms in advanced materials.

## 1. Introduction

Additive manufacturing (AM) has revolutionized component fabrication through computer-aided design and layer-wise material deposition, offering advantages such as design flexibility, cost efficiency, and rapid prototyping for aerospace, automotive, and biomedical applications [1,2,3,4]. Among AM-processed materials, Inconel 718 alloys are widely used in critical components like turbine blades and nuclear structures due to their high-temperature resistance. However, fatigue failure caused by internal defects (e.g., porosity, unmelted particles, and lack-of-fusion voids) remains a major challenge, often leading to catastrophic economic losses [5,6,7]. These defects act as stress concentrators, significantly reducing the very-high-cycle fatigue (VHCF) life of AM components [6].

To address this issue, two dominant approaches have emerged: physics-based modeling and data-driven machine learning (ML). Classical physics models, including Mayer’s model Equation (1) [8,9], the Z-parameter model Equation (3) [10], and Wang’s model Equation (2) [11], correlate fatigue life with defect size and material hardness by treating inclusions as initial cracks. While these models provide mechanistic insights, their accuracy relies heavily on empirical parameter calibration and simplified assumptions about defect morphology [12]. Recent hybrid approaches, such as Jia-Le Fan et al.’s physics-informed neural network (PINN) [13], integrate physical equations (e.g., Z-parameter) into ML frameworks to augment limited experimental data. Nevertheless, such methods remain constrained by the inherent limitations of the underlying physical models.

(1) $${\left[\sigma {(\sqrt{area)}}^{\frac{1}{6}}\right]}^{\alpha }N_{f}=C\;(Mayer’s\;model)$$

(2) $$N_{f}=\frac{9G△K_{th}^{2}}{2E{\left(\Delta \sigma -{\Delta \sigma }_{D}^{R}\right)}^{2}a_{0}}+\frac{a_{0}^{\left(1-n/2\right)}}{C{\Delta \sigma }^{n}\beta_{1}^{n}\pi^{n/2}(n/2-1)}\;(Wang’s\;model)$$

(3) $${\left[Y\sigma {(\sqrt{area)}}^{\frac{1}{6}}D^{\beta }\right]}^{\alpha }N_{f}=C\;(Z\;parameter\;model)$$

In parallel, purely data-driven ML techniques—such as Random Forest (RF), XGBoost, and support vector regression (SVR)—have demonstrated promising results in fatigue life prediction by directly mapping defect characteristics (e.g., size, location, and morphology) to fatigue properties [14,15]. For instance, Y.W. Luo et al. [15] quantified the influence of pore size, density, and proximity to surfaces on the VHCF life of SLM Inconel 718 using experimental datasets, highlighting the critical role of defect statistics. However, ML models require large, high-quality datasets for robust training, which are often unavailable due to the time-intensive nature of fatigue testing.

The methods mentioned above, no matter it is the prediction of fatigue life based on physical models [8,9,10,11,12] or the training of machine learning models after augmenting data through physical models [13], both methods encounter difficulties in building fatigue datasets and are constrained by the parameters of the physical model. Therefore, to explore the relationship between fatigue life and other parameters not encompassed by the existing physical model, it is necessary to re-optimize the established physical model. However, this is a complex task despite the existence of various machine learning methods designed to study complex fatigue behavior involving multiple parameters [13,14,15,16].

The model training for machine learning is still limited to the construction of fatigue dataset. Deep Generative Models (DGMs) are a class of machine learning models [16,17,18] that aim to learn the probability distribution of data, thereby generating new samples that resemble the training data. DGMs have widely application in fields such as image synthesis [19], natural language processing [20], and the lifetime estimation of mechanical parts [21]. This paper introduces DGMs to tackle the challenge of insufficient data and to enhance the accuracy of machine learning models [22]. Specifically, the study employed generative adversarial networks (GANs) and variational auto-encoders (VAEs) for the purpose of data augmentation. GANs comprise a generator and a discriminator; the former produces realistic samples while the latter distinguishes between real and generated samples [23]. GAN variants include CTGAN [24], CopulaGAN [25], TGAN [26], and MedGAN [27,28]. Conversely, VAEs integrate the probability distribution into the potential space during training, producing samples that are more interpretable [29]. In this work, DGMs are introduced to establish a fatigue life model for machine learning based on an existing smaller dataset.

## 2. Materials and Methods

### 2.1. Original Dataset Construction

The material under investigation in this study is additively manufactured Inconel 718 that was produced via Selective Laser Melting (SLM). As a widely used nickel-based superalloy, Inconel 718 exhibits exceptional high-temperature performance and has been extensively applied in mission-critical aerospace components, power generation systems, and automotive turbochargers [30,31,32]. The fabrication process employed an EOS M290 SLM (Krailling, Germany) system equipped with a 400 W fiber laser, utilizing high-purity argon as the shielding gas to prevent oxidation. The feedstock material consisted of gas-atomized Inconel 718 powder with a spherical morphology and controlled particle size distribution (15–45 μm). Prior to manufacturing, the substrate underwent sequential surface preparation including abrasive grinding, ultrasonic cleaning in ethanol, and vacuum drying to ensure interfacial integrity.

**Figure 1:**
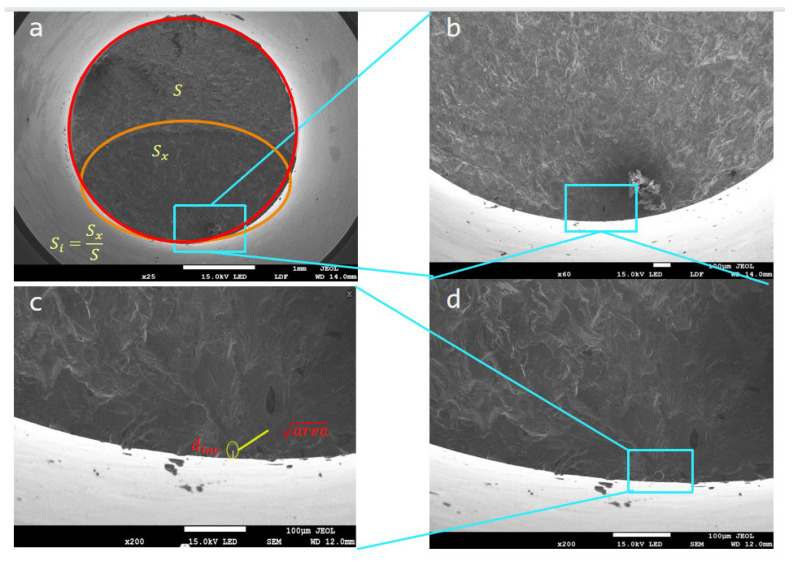
Definition of defect parameters: (**a**) ratio of crack source extension area to specimen area; (**b**,**d**) defect location; (**c**) defect parameters.

Cylindrical specimens (10 mm diameter × 80 mm length) were fabricated using a bidirectional scanning strategy with 90° interlayer rotation to minimize anisotropic effects. The optimized SLM parameters were as follows: laser power = 260 W, scan speed = 1000 mm/s, hatch spacing = 0.11 mm, and layer thickness = 40 μm. The post-processing heat treatment followed a three-stage protocol: (1) solution treatment at 950–980 °C for 1 h with air cooling, (2) aging at 720 ± 5 °C for 8 h, and (3) furnace cooling at 50 °C/h to 620 ± 5 °C, followed by 8 h isothermal holding and final air cooling [33]. The chemical composition (Table 1) and microstructural characteristics (Figure 1).

Very-high-cycle fatigue (VHCF) testing was conducted under fully reversed loading (R = −1) using ultrasonic fatigue instrumentation, with compressed air cooling employed to maintain the specimen temperature during high-frequency cycling. Fractographic analysis via scanning electron microscopy (SEM) was used to quantify critical defect characteristics, including (1) critical inclusion size (√area), (2) subsurface defect depth, and (3) crack initiation zone ratio. The experimental dataset (Table 2) and corresponding fracture surface morphologies (Figure 1) were extracted from reference [34] with validation through comparative analysis.

The very-high-cycle fatigue life experimental data and scanning electron microscope (SEM) plots for SLM were obtained from the literature [34] as shown in Table 2. Fatigue fracture analysis results of specimens subjected to ultrasonic fatigue experiments at a stress ratio of −1 were obtained including stress amplitude (σa), fatigue life (Nf), defect diameter d, defect size (square root of inclusions area √area), and inclusion distance (the shortest distance from the center of the inclusions to the surface of the specimen. dinc) and are shown in Figure 1. This newly introduced parameter $S_{i}$ (ratio of crack source extension area to specimen area) and other parameters are shown in Table 2.

### 2.2. Generative Adversarial Network (GAN) Data Expansion

As one of the most representative generative models within Deep Generative Models (DGMs), this study utilized the TensorFlow Keras Sequential API to define the neural network architectures of both the generator and the discriminator. The discriminator accepts data as input and outputs the probability of its authenticity. The GAN model is constructed by integrating the aforementioned functions of the generator and discriminator. It is compiled utilizing the binary cross-entropy loss function and the Adam optimizer. Training of the GAN involves loop iterations, where initially, the discriminator is trained on real data, followed by generated data, to effectively distinguish between the two. The generator, accepting Gaussian-distributed noise Z, aims to produce increasingly realistic data to deceive the discriminator. Upon completion of training, the generator is employed to generate. The objective function for the newly developed generative adversarial network (GAN) targeting synthetic data generation is articulated in Equation (4).

(4) $${min}_{G}{max}_{D}V(D,G)=E_{X\sim Pdata(x)}[logD(x)]+E_{Z\sim P_{z}(z)}[log(1-D(G(z)))]$$

where G is the parameter of the generator, whose objective is to generate samples that look similar to the real data. D is the parameter of the discriminator. The goal of the discriminator is to give a high probability for the real data and a low probability for the generated data to discriminate whether the samples generated by the generator are real data or not. $E_{X\sim Pdata(x)}[logD(x)]$ this term represents the samples x sampled from the real data distribution p_data(x), and then calculates the logarithm of the discriminator’s predicted probability for these real samples. The goal of the generator is to maximize the discriminator’s predicted probability for the generated samples as being real.

(5) $$E_{Z\sim P_{z}(z)}[log(1-D(G(z)))]$$

**Figure 2:**
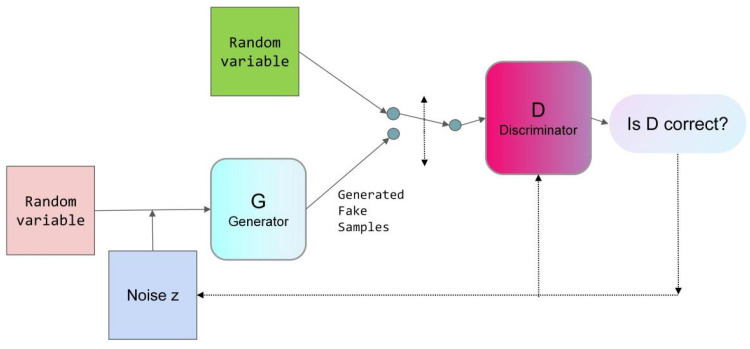
Structure of GAN model.

This term represents the input distribution of the generator. p_z (z) represents the noise sampled in the generator, which is then fed into the generator to generate a sample, and then the generated sample is fed into the discriminator. Finally, the logarithm of the discriminator’s predicted probability of these generated samples is calculated. The goal of the generator is to maximize this term so that the generated samples are harder for the discriminator to recognize as generated rather than real. The structure of the GAN is shown in Figure 2.

### 2.3. Variational Auto-Encoder (VAE) Data Expansion

**Figure 3:**
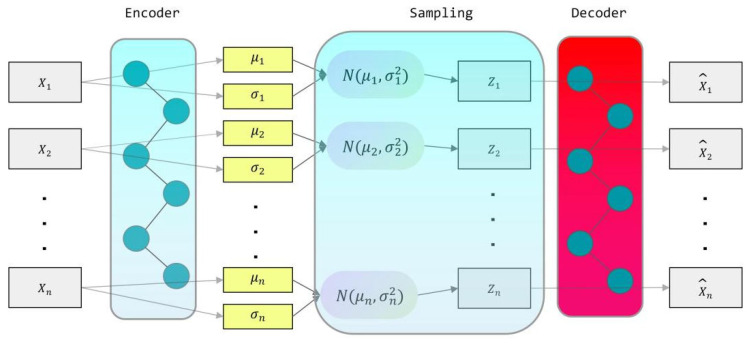
Structure of VAE model.

This study utilized TensorFlow’s Keras Sequential API to define the encoder and decoder architectures and compile the model using the Adam optimizer. The encoder processes input data through a series of dense layers to derive the mean and log variance, subsequently sampling the latent space based on these parameters. Subsequently, the decoder receives samples from the latent space to generate the output. A variational auto-encoder (VAE) model connects the encoder and decoder via a fully connected layer. Sampling of the latent space occurs through a dedicated sampling layer to facilitate new data generation. The sampling mechanism utilizes the mean and log variance to calculate the random bias within the Gaussian distribution. The VAE’s objective function comprises two components, the first being the reconstruction loss, with Mean Squared Error (MSE) serving as the reconstruction loss, as delineated in Equation (6). The second component is the KL (Kullback–Leibler) divergence, which measures the discrepancy between the distribution in the latent space and the standard normal distribution, thereby guiding the learned distribution to approximate the standard normal distribution. This objective function aims to minimize the cumulative sum of these two components. The overall objective function, formulated as the Evidence Lower Bound (ELBO), is defined in Equation (7). The aim is to optimize the ELBO for improved data representation and to ensure meaningful latent variable distributions via KL divergence constraints.

(6) $$MSE=\frac{1}{N}\sum_{i=1}^{N}{\left(x_{i}-{\hat{x}}_{i}\right)}^{2}$$

(7) $$ELBO=\frac{1}{N}\sum_{i=1}^{N}{\left(x_{i}-{\hat{x}}_{i}\right)}^{2}+-\frac{1}{2}\sum_{i=1}^{d}\left(1+log(\sigma_{i}^{2})-\mu_{i}^{2}-\sigma_{i}^{2})\right)$$

where $\frac{1}{N}\sum_{i=1}^{N}{\left(x_{i}-{\hat{x}}_{i}\right)}^{2}$ is the reconstruction error calculated from the mean square error, N is the sample size, $x_{i}$ is the original data, and ${\hat{x}}_{i}$ is the ta generated by the decoder, $-\frac{1}{2}\sum_{i=1}^{d}\left(1+log(\sigma_{i}^{2})-\mu_{i}^{2}-\sigma_{i}^{2})\right)$ is the KL scatter, d is the dimension of latent variables, and $\mu_{i}$ and $\sigma_{i}$ are the mean and variance of the latent variables, respectively. The VAE structure is shown in Figure 3. 

## 3. Development of Machine Learning Models

### 3.1. Data Usability

**Figure 4:**
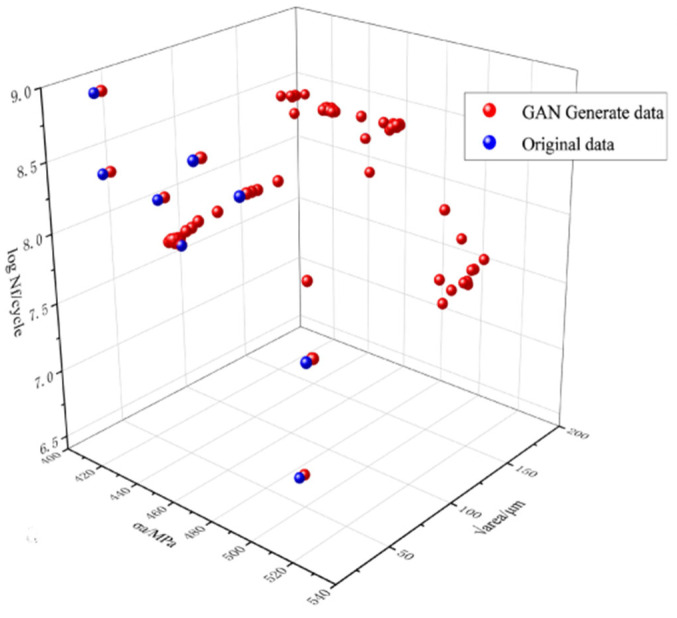
Distribution of GAN-generated data.

**Figure 5:**
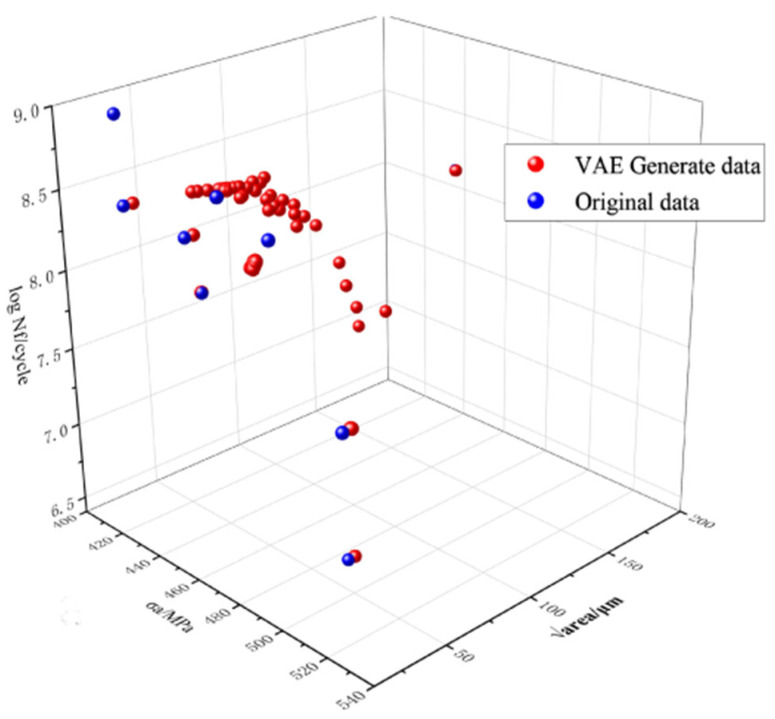
Distribution of VAE-generated data.

Utilizing the aforementioned methods, the GAN and VAE were expanded to generate 150 datasets each, as illustrated in Figure 4 and Figure 5. Prior to training the ML model, it is crucial to assess the consistency and reliability of the extended data. To validate the usability of the generated data and gain a deeper understanding of how the characteristics of the defect distribution in the specimen affect fatigue life, the Pearson correlation coefficient (PCC) was employed to quantify the relationship between individual defect characteristics and fatigue life.

(8) $$P=\frac{\sum \left(X_{i}-\bar{X}\right)(Y_{i}-\bar{Y})}{\sqrt{\sum {\left(X_{i}-\bar{X}\right)}^{2}\sum {\left(Y_{i}-\bar{Y}\right)}^{2}}}$$

where X_i_ and Y_i_ are the values of two characteristic variables, and $\bar{X}$ and $\bar{Y}$ are the means of two characteristic variables.

**Figure 6:**
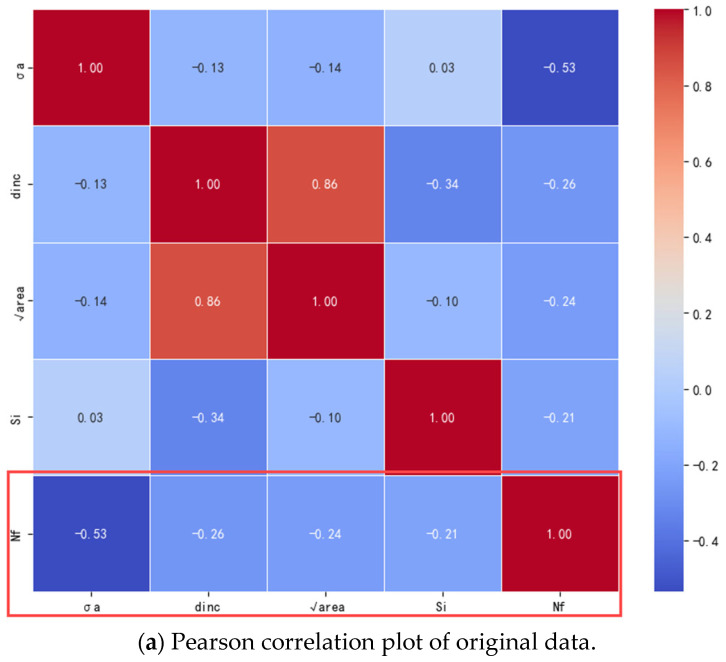

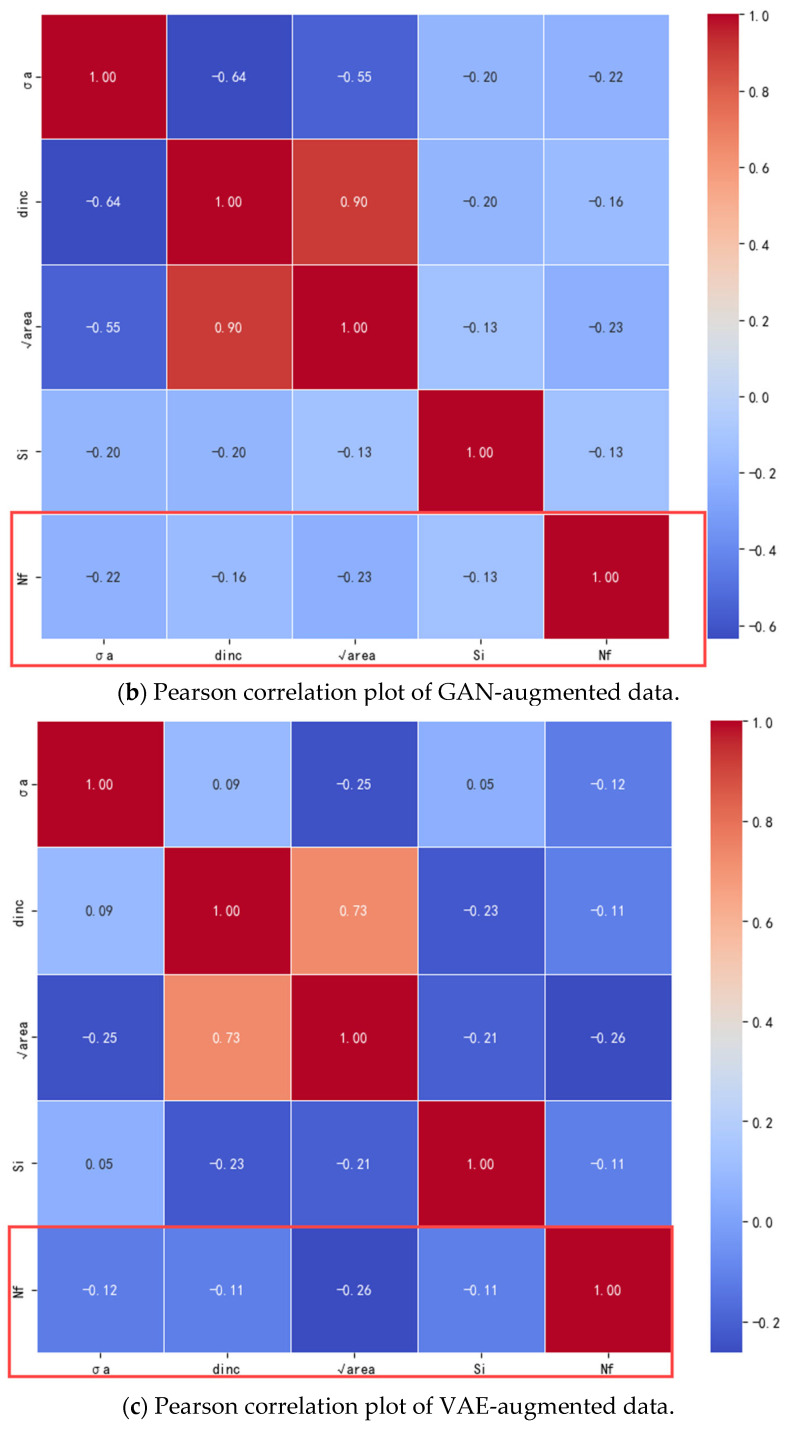
Pearson correlation plot.

The original and extended datasets were imported to calculate the Pearson correlation coefficients, and PCC heat maps between the fatigue life and the relevant parameters for each dataset were obtained, as shown in Figure 6. The similarity between the extended datasets in Figure 6b,c and the original dataset in Figure 6a demonstrates the reliability of the data generated by GAN and VAE. It is reasonable to build the expanded dataset using these two generative models.

### 3.2. Data Preprocessing

With the aforementioned methods, 150 sets of datasets were obtained using the GAN and VAE, each comprising five variables: σa/Mpa, dinc/μm, and √area/μm, where √area is derived from the generated d. Additionally, the ratio of the extended area to the specimen’s area Si and Nf/cycle have different dimensions and units, affecting the uniformity and potentially causing escalated training duration and convergence challenges. Consequently, preprocessing of the dataset prior to its utilization in the ML model for training is imperative. This process aims to mitigate dimensional complexity and redundancy within the data. The normalization in Equation (9) was employed for this purpose. In addition, the logarithm of fatigue life was utilized for both model training and testing, as depicted in Equation (10).

(9) $$X_{norm}=\frac{X-X_{min}}{X_{max}-X_{min}}$$

where X_norm_, X, X_min_, and X_max_ represent the normalized, original, minimum, and maximum values of the variable X, respectively.

(10) $$N_{log}=logN_{f}$$

where N_log_ and N_f_ denote the logarithmic and original values of the fatigue life, respectively.

It is imperative to ensure that the established machine learning models possess both reliability and generalization capabilities. The extended dataset was divided in a 7:3 ratio between the training and validation sets, with the original data serving as the test set and was not included in the training process. The training set is utilized for the iterative training of the model, aiming to develop a fatigue life prediction model, while the validation set facilitates hyperparameter optimization to select the optimal hyperparameter. Furthermore, to mitigate the impact of data randomness on the prediction outcomes, 5-fold cross-validation is implemented on the training data. Finally, the original data excluded from model training is fed into the trained model for prediction, enabling the evaluation of the model’s prediction performance.

### 3.3. Machine Learning Models

**Figure 7:**
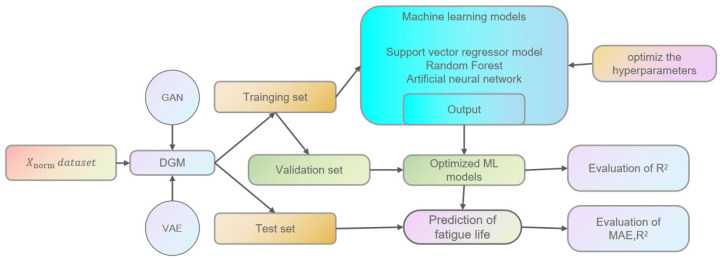
Framework of the modeling process for fatigue life prediction.

Following data expansion and preprocessing, the training set is utilized for ML model training. Subsequently, upon hyperparameter optimization, the optimal ML models are obtained, and the R^2^ value of the validation set within the training set is computed to assess the fit of the ML models. This is followed by fatigue life prediction using the optimal ML models, with R^2^ and MAE serving as metrics to evaluate the model’s generalization ability and accuracy. The process is illustrated in Figure 7.

#### 3.3.1. Support Vector Regression Model

Following data expansion and preprocessing, the data are input into the Support Vector Regression (SVR) model for training, where the objective is to identify an optimal hyperplane that minimizes the model’s prediction error on the training data while tolerating a certain level of error [34]. The training is an optimization problem, wherein the penalty parameter (*C*), the kernel function parameters (*γ*), and the tolerance (*ε*) are fine-tuned using methods like cross-validation. This phase aims to evaluate the model’s performance on validation data and select the optimal hyperparameter configuration to prevent overfitting or underfitting. The available kernel functions include Linear Kernel (linear), Radial Basis Function (RBF), Polynomial Kernel (poly), and Sigmoid Kernel (sigmoid). The regression function is presented in Equation (11) [35].

(11) $$f(x)=\sum_{i=1}^{n}(\alpha_{i}-\alpha_{i}^{*})\cdot K(x_{i},x_{j})+b,\alpha_{i},\alpha_{i}^{*}\in \left[0,C\right]$$

where $\alpha_{i}$ and $\alpha_{i}^{*}$ are the Lagrange multipliers, b is the bias, and C is the penalty parameter. $K(x_{i},x_{j})$ denotes the kernel function distribution [36] with radial kernel (rbf), linear kernel, and polynomial kernel [36].

In the SVR structure, σa (MPa), dinc (μm), √area (μm), and Si are used as the x input parameters, while life is employed as the f(x) output dependent variable.

#### 3.3.2. Random Forest Model

The training phase of the RF regression model constructs multiple uncorrelated decision trees. In this study, σa/Mpa, dinc/μm, √area/μm, and Si were used as input parameters, and the lifetime was used as the $y_{RF}$ output dependent variable. The training dataset is divided into multiple sub-datasets. Different sub-datasets are used to train different decision trees, denoted as DTk, where k is a natural number. For each decision tree, the predicted output vector is calculated. Finally, the result is obtained by averaging the predicted output vectors of all decision trees, as shown in Equation (12) [37].

(12) $$y_{RF}=\frac{1}{k}\sum_{p=1}^{k}y_{p}^{pre}$$

There are four important parameters in the RF model, including nestimators, min_samples_split, min_samples_leaf, and max_depth. Grid hyperparameter optimization is used for parameter optimization of the RF parameters.

#### 3.3.3. Artificial Neural Network

Artificial neural networks (ANNs) are composed of numerous neurons, which include an input layer for predicting outcomes. The input layer receives the input data, with each node representing an input feature. The hidden layers facilitate the learning of complex data relationships. There may be multiple hidden layers, each composed of numerous nodes. The output layer, where each node corresponds to an output category or value, produces the predicted results [38]. The neuron is the fundamental unit of an ANN. The input data passes through the input layer and propagates through the hidden layers in sequence, eventually producing results in the output layer. Each node in the hidden layers receives input values from the nodes of the previous layer. These input values are then processed by weights, biases, and activation functions to generate an output, which is finally transmitted to the next node. The regression function yi of the i-th neuron is expressed by the following Equation (13) [39,40,41,42].

(13) $$y_{i}=f_{i}\left(\sum \omega_{i,j}x_{j}-t_{i}\right)$$

where $\omega_{i}$_,j_ is the weight of the neural network, x_j_ denotes the input of the current neuron node, f_j_ is the activation function, and t_i_ is the threshold parameter.

The general idea is to use a training dataset and continuously adjust the weights through backpropagation until the model is able to accurately predict the input data. Commonly used optimization algorithms include gradient descent, stochastic gradient descent, etc. The weights are updated at each iteration to minimize the error function. The error function is given in Equation (14) [39,40,41,42].

(14) $$E=\frac{1}{2}\sum_{i=1}^{n}\left\|y_{i}-y_{i}^{true}\right\|$$

where E is the error function, n is the number of samples in the dataset, $y_{i}$ is the actual output of the regression function, and $y_{i}^{true}$ is the true value of the fatigue life of the experimental fatigue test. In this study, a multilayer perceptron (MLP) artificial neural network regressor was used.

#### 3.3.4. Model Evaluation Criteria

To evaluate the accuracy of the ML model in predicting fatigue life, the coefficient of determination R^2^ and the mean absolute error (MAE) were used. R^2^ is an accurate representation of the degree of fit of the regression model, with values close to 1 reflecting a high accuracy of the model and good fit. The MAE calculates the mean absolute error of the fatigue predicted life and the fatigue experimental life, with values close to 0 indicating high model accuracy. The formulas of $R^{2}$ and MAE are described in Equations (15) and (16), respectively.

(15) $$R^{2}(y,y^{pre})=1-\frac{\sum_{i=1}^{n}{\left(y_{i}^{true}-y_{i}^{pre}\right)}^{2}}{\sum_{i=1}^{n}{\left(y_{i}^{true}-y_{mean}\right)}^{2}}$$

(16) $$MAE(y,y^{pre})=\frac{1}{n}\sum_{i=1}^{n}\left|y_{i}^{true}-y_{i}^{pre}\right|$$

where $y_{i}^{true}$ denotes the *i*-th original data, $y_{i}^{pre}$ denotes the *i*-th predicted data, and $y_{mae}$ is the average of the original data.

### 3.4. Hyperparameter Optimization

The machine learning model training process adjusts the hyperparameters of the model to improve performance and generalization. Hyperparameters are parameters that are set before the model is trained and their values cannot be learned through the training process and need to be adjusted manually. The choice of hyperparameters is crucial for the performance and generalization ability of the model. In order to accurately assess model performance with different hyperparameter settings, cross-validation is often used. The training data are divided into training and validation sets. The training set is used to iteratively train the model, while the validation set assesses the quality of the model’s training. Prior to the optimization process, it is necessary to set the range of values for the hyperparameters and parameters, as well as to select the method for optimizing the hyperparameters, e.g., lattice search [14], stochastic search, and Bayesian optimization [24]. The optimization set for each ML model is described in Table 3.

For the support vector regression model, three hyperparameters *C*, *ε*, *γ* and kernel function selection were optimized. These hyperparameters are crucial for the accuracy and generalization ability of the model. In this study, the penalty parameter C takes 15 values, the ε and the coefficients *γ* of the kernel function take 5 values in the range of C ϵ [10, 20, ……140, 150], *ε* ϵ [0.001, 0.01, 0.1, 1, 10], and *γ* ϵ [0.001, 0.01, 0.1, 1, 10].

Four parameters were optimized for the RF model, namely the total number of trees (n_estimators ϵ [50, 100, 150, 200, 500]), minimum number of samples for node splits (min_samples_split ϵ [2, 4, 6, 8, 10, 16]), minimum number of samples for leaf nodes (min_samples_leaf ϵ [1, 2, 4]), and the maximum depth of the tree (max_depth ϵ [None, 10, 20, 30, 40, 50]).

For the ANN model, the following four parameters were identified as key hyperparameters of the neural network model. Hidden_layer_sizes are defined as a list of tuples, where each tuple specifies the number and size of the hidden layers. For instance, (20) signifies a single hidden layer comprising 20 neurons. Utilizing list comprehension, configurations such as [(*i*, *j*, *k*)] indicate attempts at varying hidden layer sizes with respective neuron counts. The ranges for these configurations are as follows: *i* ϵ [20, 30...70, 80] for layer sizes, and *j*, *k* ϵ [0, 10, 20, 40] and [0, 10, 15, 20] for neuron counts and subsequent layers, respectively. The activation parameter specifies the activation function, which introduces non-linearity into each hidden layer, with the options ‘identity’ (17), ‘RELU’ (18), ‘sigmoid’ (19), and ‘tanh’ (20). The solver parameter was the chosen optimizer for the model, with options such as ADAM, LBFGS, and SGD. Lastly, max_iter defines the maximum number of iterations or training rounds for the model, after which, training will cease regardless of convergence status.

(17) Identity: (f(x)=x)

(18) RELU: (f(x)=max(0,x))

(19) Sigmoid: (f(x)=1/(1+exp(−x)))

(20) $$\mathrm{Tan}h:\;(f(x)=\frac{e^{x}-e^{-x}}{e^{x}+e^{-x}})$$

## 4. Results and Discussion

Three ML models, SVR, RF, and ANN, constructed based on deep generative models were developed to predict the fatigue life of Inconel 718 using the two extended datasets mentioned in Section 2.2 and Section 2.3. The initial prediction accuracies of the ML models on the training set were compared. Subsequently, the prediction results of the different models were juxtaposed with the original data to evaluate their performances. These models are denoted by a combination of the deep learning model and machine learning model sizes used for data augmentation in Table 4 and Table 5; for instance, GAN-SVR denotes the SVR model trained on the GAN extended dataset.

### 4.1. Parameter Configuration and Training Set Performance of the ML Model

In this study, the hyperparameters of the GAN-SVR model are optimally configured for the kernel function using the radial basis function (RBF) (21):

(21) $$K(x_{i},x_{j})=exp(-\gamma {\left\|x_{i}{-x}_{j}\right\|}^{2})$$

where *γ* is the nuclear parameter in the optimal configuration of hyperparameters for GAN-SVR.

The validation set performance, with each calculated R^2^ value, is listed in Table 6. Based on the provided graph illustrating the fatigue life validation set prediction results of the ML model and R^2^, the optimized model can be obtained. The GAN-ANN model (R^2^ = 0.965) exhibited superior performance compared to the GAN-SVR model (R^2^ = 0.965) and GAN-RF model (R^2^ = 0.965). Meanwhile, the VAE-RF model (R^2^ = 0.983) exhibited superior performance compared to the VAE-ANN model (R^2^ = 0.969) and VAE-SVR model (R^2^ = 0.979).

**Figure 8:**
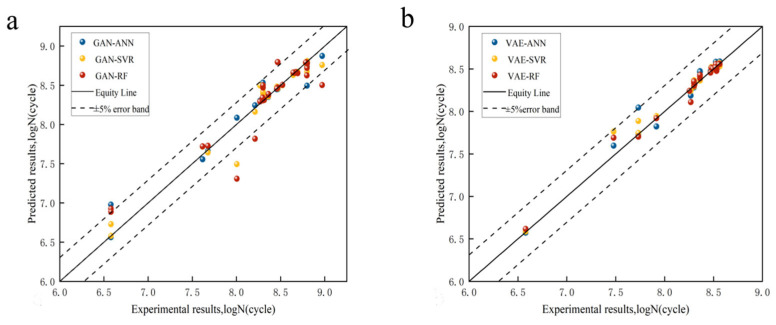
(**a**) GAN models’ predictive accuracy; (**b**) VAE models’ predictive accuracy.

A comparison of the results of the fatigue life calculated by the optimized ML model (with tuned hyperparameters and parameters) and the experimental results of the validation set is shown in Figure 8.

### 4.2. Fatigue Life Prediction

**Figure 9:**
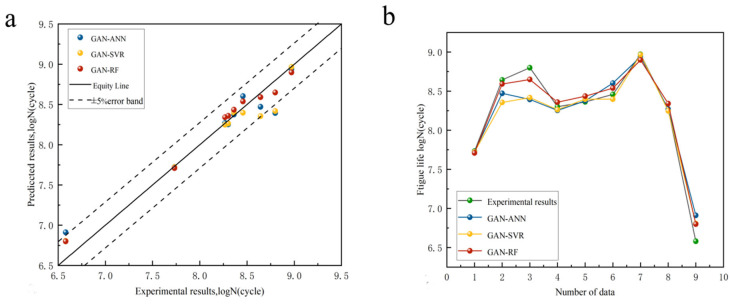
(**a**) GAN models’ predictive accuracy; (**b**) variation in GAN models’ predicted life compared to experimental data.

**Figure 10:**
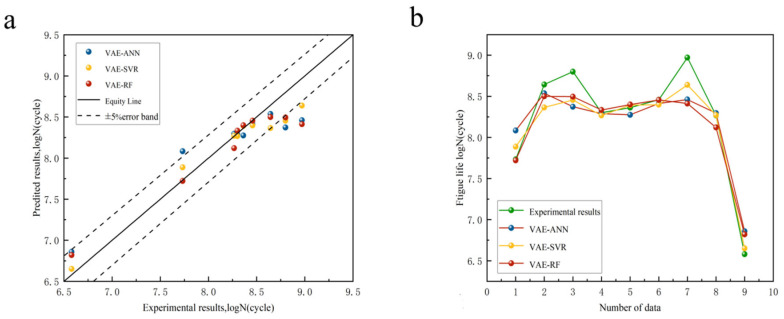
(**a**) VAE models’ predictive accuracy; (**b**) variation in VAE models’ predicted life compared to experimental data.

The validation set model and the hyperparameter-optimized ML model were compared and finally the optimized model was used to predict the fatigue life using the original dataset to evaluate the generalization ability of the model. The prediction accuracies of the GAN-ML models and VAE-ML models are shown in Figure 9 and Figure 10. The R^2^ and MAE are presented in Table 7. Based on the values of R^2^, the GAN-ANN model after GAN data enhancement performed worse (R^2^ = 0.919, MAE = 1.62%) than the GAN-SVR model (R^2^ = 0.929, MAE = 1.46%) and GAN-RF model (R^2^ = 0.975, MAE = 1.13%). The VAE-ANN model based on enhanced VAE data performed worse (R^2^ = 0.856, MAE = 2.06%) than the VAE-SVR model (R^2^ = 0.917, MAE = 1.61%) and VAE-RF model (R^2^ = 0.891, MAE = 1.57%). It can be observed from Figure 10 that the data predicted by the VAE-ML model fell outside the error range in the higher cycle ranges, clearly indicating that the model performed poorly when predicting data in these higher cycle ranges.

The analysis indicated that, in the validation set, the GAN-based machine learning models (GAN-ML) demonstrated lower performance compared to the VAE-based machine learning models (VAE-ML). However, in the test set, the GAN-ML models significantly outperformed the VAE-ML models. The accuracy of the GAN-ML models in the test set was not only higher than that of the VAE-ML models but was also comparable to their performance in the validation set. This consistent performance indicates the superior generalization capabilities of the GAN-ML models. In contrast, although the VAE-ML models showed higher accuracy in the validation set, they were unable to maintain this advantage in the test set. These data suggest that GAN-ML models are better at handling unseen data, reflecting their enhanced generalization capabilities compared to VAE-ML models. While the GAN-RF models exhibited significant underfitting and deliver inferior performance in the validation set, they nevertheless attained a higher R^2^ score with fewer training sets, which, in turn, makes GAN-ANN and GAN-SVR models appear more reliable by comparison.

## 5. Conclusions

This study proposes an extensible fatigue life prediction framework for high-temperature nickel-based alloys (demonstrated on Inconel 718) by systematically integrating defect characterization and generative modeling. Fatigue data were derived from SEM-based fracture analysis, defect size/location measurements, and a novel parameter—the fatigue crack ductile zone-to-specimen area ratio—to quantify crack growth behavior. To address data limitations, deep generative models (DGMs), including GANs and VAEs, were employed for data augmentation, followed by Pearson’s correlation analysis to validate parameter–life relationships and ensure the physical consistency of the synthesized datasets. The framework combines advanced experimental characterization, probabilistic data generation, and statistical validation to enhance predictive accuracy in fatigue life assessment.

The volume and quality of the initial dataset critically influence the performance of GAN and VAE models in fatigue life prediction, as observed in this study. A limited data volume restricts the generative models’ ability to capture the full statistical distribution of defect characteristics and loading conditions, leading to suboptimal synthetic data diversity. For instance, the GAN-RF model exhibited underfitting, likely due to insufficient coverage of defect distance interactions in the original dataset. Data quality issues, such as imbalanced defect size ranges or measurement uncertainties in SEM-derived parameters propagate into the generated data, amplifying biases in downstream ML predictions, as evidenced by the VAE-ANN’s overfitting (training MAE = 1.62% vs. test MAE = 2.46%). Future work should prioritize expanding the experimental dataset with controlled defect geometries and inclusion distributions while integrating uncertainty quantification to decouple measurement noise from true physical trends.

The comparative performance analysis of machine learning (ML) models integrated with generative adversarial network (GAN) and variational autoencoder (VAE) frameworks revealed distinct trends: the GAN-based models consistently outperformed their VAE counterparts, with GAN-RF achieving the highest predictive accuracy (R^2^ = 0.975) and minimal error (MAE = 1.13%), followed by GAN-SVR (R^2^ = 0.929, MAE = 1.46%) and GAN-ANN (R^2^ = 0.919, MAE = 1.62%). In contrast, the VAE-based models exhibited comparatively lower performance, with VAE-SVR (R^2^ = 0.865, MAE = 2.00%), VAE-RF (R^2^ = 0.879, MAE = 2.01%), and VAE-ANN (R^2^ = 0.861, MAE = 2.46%) demonstrating reduced generalization capability. These results highlight the superior robustness of GAN-enhanced models in fatigue life prediction, particularly GAN-RF, which delivered the highest R^2^ and lowest MAE, while the VAE-driven models exhibited larger error margins, suggesting limitations in their ability to generalize across unseen datasets.

The results showed that the VAE-ML models outperformed the GAN-ML models on the validation set, but on the test set, the VAE-ANN (validation set R^2^ = 0.923 vs. test set R^2^ = 0.861) and VAE-RF (validation set R^2^ = 0.968 VS test set R^2^ = 0.879) models showed overfitting. Overfitting occurs when a model achieves high performance on training data but significantly underperforms on new data, primarily due to excessively capturing noise or localized patterns in the data, thereby weakening its generalization capability. The GAN-ML models demonstrated better performance than the VAE-ML models in terms of R2 and MAE, indicating a better fit.

The GAN-RF (test set R^2^ = 0.864) models experienced underfitting. Underfitting occurs when a model exhibits poor performance on both training and test data, typically stemming from an oversimplified architecture or insufficient learning capacity, which prevents it from capturing fundamental patterns in the dataset. The GAN-SVR, GAN-ANN, and VAE-SVR models might be better for predicting fatigue life.

Furthermore, the purely data-driven models employed in this study lack physics-constrained interpretability, limiting their ability to align predictions with established mechanical principles or to generalize across untested operational conditions. Therefore, developing hybrid physics-informed ML frameworks that explicitly couple defect propagation laws with data-driven models could improve interpretability.

## Figures and Tables

**Table 1:** Chemical composition of Inconel 718 alloy (wt.%).

Ni	Nb	Mo	Ti	Al	Cr	C	Fe
53.00	5.30	3.00	1.00	0.50	19.00	0.05	Balance

**Table 2:** Fatigue experiment data for Inconel 718.

$\sigma_{a}/Mpa$	d/μm	$d_{\mathrm{inc}}/\mu m$	$\sqrt{\mathrm{area}}/\mu m$	$S_{i}/1$	$N_{f}/Cycle$
522	21.00	8.00	18.61	0.34	5.4 × 10^7^
493	21.00	3.00	18.61	0.29	4.4 × 10^8^
474	17.00	3.00	15.06	0.29	6.3 × 10^8^
474	6.00	6.00	5.31	0.27	2 × 10^8^
444	32.85	128.57	29.11	0.34	2.3 × 10^8^
420	25.45	15.90	22.55	0.31	2.87 × 10^8^
415	28.81	141.30	25.53	0.27	9.35 × 10^8^
452	200.00	430.76	177.24	0.29	1.85 × 10^8^
496	58.33	333.32	51.69	0.25	3.8 × 10^6^

**Table 3:** Hyperparameters and parameters of the considered ML models.

ML Model	Tuning Entity	Range	No. of Values
SVR	*K*	[‘linear’, ‘poly’, ‘rbf’, ‘sigmoid’]	4
	*C*	[10, 20, ……, 140, 150]	15
	*ε*	[0.001, 0.01, 0.1, 1, 10]	5
	*γ*	[0.001, 0.01, 0.1, 1, 10]	5
RF	n_estimators	[50, 100, 150, 200, 500]	5
	max_depth	[None, 10, 20, 30, 40, 50]	6
	min_samples_split	[1, 2, 4, 6, 8, 10, 16]	6
	min_samples_leaf	[1, 2, 4]	6
ANN	hidden_layer_sizes	[(*i*, *j*, *k*)] *i* ϵ [20, 30, …, 70, 80] *j* ϵ [0, 10, 20, 40] *k* ϵ [0, 10, 15, 20]	(7, 5, 4)
	activation	[identity, RELU, sigmoid, tanh]	4
	solver	[adam, lbfgs, sgd]	3
	max_iter	[10, 50, 100, 500, 1000, 5000]	6

**Table 4:** Values of hyperparameters of GAN-ML models.

ML Model	Tuning Entity	Value
GAN-SVR	*K*	RBF
	*C*	100
	*ε*	0.01
	*γ*	1
GAN-RF	n_estimators	100
	max_depth	20
	min_samples_split	1
	min_samples_leaf	2
GAN-ANN	hidden_layer_sizes.	(20, 20, 5)
	activation.	[tanh]
	solver.	[lbfgs]
	max_iter.	500

**Table 5:** Values of hyperparameters of VAE-ML model.

ML Model	Tuning Entity	Value
VAE-SVR	*K*	RBF
	*C*	20
	*ε*	0.01
	*γ*	1
VAE-RF	n_estimators	100
	max_depth	20
	min_samples_split	1
	min_samples_leaf	2
VAE-ANN	hidden_layer_sizes.	(80, 10, 20)
	activation.	[tanh]
	solver.	[lbfgs]
	max_iter.	100

**Table 6:** Prediction accuracy of the optimized ML model for the validation set.

ML Model	R^2^
GAN-SVR	0.958
GAN-RF	0.864
GAN-ANN	0.965
VAE-SVR	0.979
VAE-RF	0.968
VAE-ANN	0.923

**Table 7:** Prediction accuracy of the optimized ML model on the test set.

ML Model	R^2^	*MAE* (Per Cent)
GAN-SVR	0.929	1.46
GAN-RF	0.975	1.13
GAN-ANN	0.919	1.62
VAE-SVR	0.865	2.00
VAE-RF	0.879	2.01
VAE-ANN	0.861	2.46

## Data Availability

Data will be made available on request.
